# 

**Published:** 1902-04

**Authors:** 


### BOOKS RECEIVED.

Diseases of the Intestines. Their Special Pathology, Diagnosis and Treatment. With sections on Anatomy and Physiology, Microscopic and Chemic Examination of the Intestinal Contents, Secretions, Feces and Urine; Intestinal Bacteria and Parasites; Surgery of the Intestines; Dietetics; Diseases of the Rectum, etc. By John C. Hemmeter, M. D., Philos. D., Professor in the Medical Department of the University of Maryland; Consultant to the University Hospital and Director of the Clinical Laboratory; Author of a treatise on "Diseases of the Stomach," etc. In two volumes. Vol. II. Appendicitis, Tuberculosis, Syphilis, Actinomycosis of Intestine, the Occlusions, Contusions, Rupture, Enterorrhagia, Intestinal Surgery, Atrophy, Abnormalities of Form and Position, Thrombosis, Embolism, Amyloidosis, Neuroses of the Intestines, Intestinal Parasites, Diseases of Rectum. With plates and many other original illustrations. Octavo, 675 pages. Philadelphia: P. Blakiston's Son & Co. 1902. [Price, Vol. II., $5.00 net; set complete, $10.00.]

Quiz Compends. Compend of General Pathology. By Alfred Edward Thayer, M. D., Assistant Instructor in Gross Pathology, Cornell Medical College; Pathologist to the City Hospital. Containing 78 illustrations. Philadelphia: P. Blakiston's Son & Co. 1902. [Price, 80 cents net.]

Syphilis. A Symposium of Special Contributions from Seventeen Different Writers. New York: E. B. Treat & Co. 1902. [Price, $1.00.]

A Manual of Gynecology for the use of Students and General Practitioners. By F. H. Davenport, A.B., M.D., Assistant Professor in Gynecology, Harvard Medical School. New (4th) edition, revised and enlarged in one 12mo volume of 402 pages, with 154 illustrations. Philadelphia and New York: Lea Brothers & Co. 1902. [Price, cloth, $1.75 net.]

Seventeenth Annual Report of the State Board of Health and Vital Statistics of the Commonwealth of Pennsylvania. Vols. I. and II. Transmitted to the Governor, November 30, 1900. Benjamin Lee, M. D., Secretary. Wm. Stanley Ray, State Printer of Pennsylvania. 1901.

The American Year-Book of Medicine and Surgery for 1902. A Yearly Digest of Scientific Progress and Authoritative Opinion in all branches of Medicine and Surgery, drawn from journals, monographs, and text-books of the leading American and foreign authors and investigators. Arranged, with critical editorial comments, by eminent American specialists, under the editorial charge of George M. Gould, A.M., M.D. In two volumes—Volume I, including General Medicine, Octavo, 700 pages, illustrated; Volume II, General Surgery, Octavo, 684 pages, illustrated. Philadelphia and London: W. B. Saunders & Co. 1902. [Price per volume: Cloth, $3.00 net; half-morocco, $3.75 net.]

A Practical Manual of Insanity. For the Student and General Practitioner. By Daniel R. Brower, A. M., M D, LL. D., Professor of Nervous and Mental Diseases in Rush Medical College, in Affiliation with the University of Chicago, and in the Post-Graduate Medical School, Chicago; and Henry M. Bannister, A. M., M. D., formerly Senior Assistant Physician, Illinois Eastern Hospital for the Insane. Handsome octavo of 426 pages, with a large number of full-page inserts. Philadelphia and London: W. B. Saunders & Company. 1902. [Cloth, $3.00 net.]

Morphinism and Narcomania from Opium, Cocain, Ether, Chloral, Chloroform, and other Narcotic Drugs; also the Etiology, Treatment, and Medicolegal Relations. By T. D. Crothers, M.D., Superintendent of Walnut Lodge Hospital, Conn., Professor of Mental and Nervous Diseases, New York School of Clinical Medicine, etc., 12mo of 351 pages. Philadelphia and London; W. B. Saunders & Co. 1902. [Cloth, $2.00 net.]

Johnson's First Aid Manual Suggestion for Prompt Aid to the Injured in Accidents and Emergencies. Edited by Fred B. Kilmer. Illustrated. Pp. 113. New Brunswick, N. J: Johnson & Johnson, 1901. [Cloth, 50 cents.]

Progressive Medicine, Vol. I., 1902. A Quarterly Digest of Advances, Discoveries and Improvements in the Medical and Surgical Sciences. Edited by Hobart Amory Hare, M. D, Professor of Therapeutics and Materia Medica in the Jefferson Medical College of Philadelphia Octavo, 452 pages, 5 Illustrations. Philadelphia and New York: Lea Brothers and Co. 1902. [Per annum, in four cloth-bound volumes, $10.00; per volume, $2.50, by express prepaid to any address.]

Report of the Commissioner of Education. For the year 1899-1900. Volume II. Washington: Government Printing Office. 1901.

Transactions of the Royal Dublin Society. Volume VII. Parts 8–13. 1900.

Proceedings of the Royal Dublin Society. Volume IX. Parts 3 and 4. 1900.

